# An algorithm to simulate nonstationary and non-Gaussian stochastic processes

**DOI:** 10.1186/s43065-021-00030-5

**Published:** 2021-06-21

**Authors:** H. P. Hong, X. Z. Cui, D. Qiao

**Affiliations:** 1 Department of Civil and Environmental Engineering, University of Western Ontario, London, N6A 5B9 Canada; 2 China Construction Eighth Engineering Division Corp, LTD., Shanghai, China

**Keywords:** Simulation, Nonstationary and non-Gaussian process, S-transform, Continuous wavelet transforms, Seismic ground motions, Wind velocity

## Abstract

We proposed a new iterative power and amplitude correction (IPAC) algorithm to simulate nonstationary and non-Gaussian processes. The proposed algorithm is rooted in the concept of defining the stochastic processes in the transform domain, which is elaborated and extend. The algorithm extends the iterative amplitude adjusted Fourier transform algorithm for generating surrogate and the spectral correction algorithm for simulating stationary non-Gaussian process. The IPAC algorithm can be used with different popular transforms, such as the Fourier transform, S-transform, and continuous wavelet transforms. The targets for the simulation are the marginal probability distribution function of the process and the power spectral density function of the process that is defined based on the variables in the transform domain for the adopted transform. The algorithm is versatile and efficient. Its application is illustrated using several numerical examples.

## Introduction

Observed time histories of the seismic ground motions [31], wind velocity [48], wave height [32], etc. fluctuate randomly in time and space. The time histories are used as the input to carry out the structural analysis. Since the available recorded time histories of the random phenomena are limited, their synthetics are generated and used in practice. The simulation is based on the theory of stochastic processes [7, 22, 25]. The simulated ground motions are used to assess seismic risk of infrastructures such as dams and portfolio of buildings (e.g., [3, 24]). The simulated winds are employed to assess the performance structures and infrastructure system [55, 60].

For stationary Gaussian processes, and evolutionary processes [38], the simulation can be carried out using the spectral representation method [23, 47], developed based on the ordinary Fourier transform (FT). A stationary process is defined by its power spectral density (PSD) function, and an evolutionary process is defined by the evolutionary PSD that is a function of an amplitude modulation function. The evolutionary process with time-dependent amplitude modulation is widely used in generating seismic ground motions [1, 10] and fluctuating wind velocity for high-intensity wind events [5, 17, 20, 21].

Masters and Gurley [27] proposed an iterative spectral correction algorithm to simulate the stationary non-Gaussian processes, where the spectral representation method is used in each iteration to generate the time history. They showed that their algorithm outperforms the SRM-based simulation techniques presented in Yamazaki and Shinozuka [58], Gurley and Kareem [15], Grigoriu [14], and Deodatis and Micaletti [11]. It is noted that an algorithm similar to the spectral correction algorithm, namely the iterative amplitude adjusted Fourier transform (IAAFT) algorithm, was proposed by Schreiber and Schmitz [43, 44] in the context of generating surrogate for statistical hypothesis testing. The use of the translation process for the stationary non-Gaussian process proposed in Grigoriu [14] was extended for the nonstationary processes by others, including Ferrante et al. [12] and Shields et al. [46].

The evolutionary PSD is often assessed using (time-dependent) windowed Fourier transform, such as the short-time Fourier transform [6]. The resolution of such a transform is controlled by the width of the window. As the width of the window increases, a better resolution is obtained at the low frequencies, and the resolution in time deteriorates. A good resolution in both time and frequency (i.e., scale) can be obtained by applying the continuous wavelet transforms (WT) [9, 35]. A procedure to estimate the evolutionary PSD by applying the continuous WT was proposed by Spanos and Failla [50]. However, an algorithm that directly applies the continuous WT to simulate the nonstationary stochastic processes with a prescribed wavelet spectrum or time-scale PSD was unavailable. Recently, an iterative algorithm was presented by Chavez and Cazelles [4] to generate surrogate for statistical hypothesis testing. We will point out, in the following sections, a potential weakness of the algorithm, as well as the link between this algorithm and an interesting way of defining nonstationary processes in the wavelet domain introduced by Maraun et al. [26]. The lack of continuous WT-based algorithm to simulate time histories is partly due to that the use of continuous WT does not lead to the decomposed signal to be represented by a set of orthogonal basis functions. Rather than using the continuous WT, the application of the discrete WT and wavelet packet transform that have orthogonal basis functions is presented in Gurley and Kareem [16], and Yamamoto and Baker [57]. The resolution obtained by using these discrete transforms is less refined than that obtained by using the continuous WT.

The phase information in WT is local, while the phase information in the Fourier transform refers to the harmonics at zero time [52]. Stockwell et al. [53] (see also [37]) developed the S-transform (ST) that provides the time-frequency representation of the analyzed signal. It is a hybrid of continuous WT and windowed FT. The S-transform provides frequency-dependent resolution. Similar to the continuous WT, ST does not lead to a decomposed signal to be represented by a set of orthogonal basis functions. Stockwell [52] proposed a discrete orthonormal S-transform. The simulation of the seismic ground motions by using the discrete orthonormal S-transform or combination with ST was presented in Cui and Hong [8] and Hong and Cui [19]. However, an algorithm by using ST alone to simulate nonstationary stochastic processes has not been reported in the literature.

There are other techniques used to simulate the nonstationary processes. These include the application of autoregressive moving-average (ARMA) [42], Karhunen–Loéve expansion [36, 49, 51], and polynomial chaos [41], and Hilbert-Huang transform [56]. The ARMA uses the recursive relations that connect the random quantity to be simulated at successive time increments. The application of the Karhunen–Loéve expansion uses the eigenfunctions that are obtained by solving Fredholm integral equation of the second kind. In the Hilbert-Huang transform, a random process is decomposed into intrinsic mode functions by the method of empirical mode decomposition. A review of these simulation procedures is beyond the present study since these techniques involve varieties of mathematical concepts and algorithms.

In the present study, we exam and extend the definition of the nonstationary processes in the transform domain. We proposed an iterative power and amplitude correction (IPAC) algorithm to simulate nonstationary and non-Gaussian processes. The algorithm could be viewed as an extension of IAAFT [43] and the spectral correction algorithm [27] and is rooted in the concept of defining the stochastic processes in the transform domain. In particular, we provide details of using the proposed algorithm with FT, ST and WT, where the energy distribution in the transform domain that satisfies energy preservation is prescribed, and the marginal probability distribution function of the process is given. We provide numerical examples to show the proposed algorithm and compare the simulated time histories obtained by using the ST-based and (continuous) WT-based approach.

## Fourier transform, S-transform, and wavelet transforms

This section summarizes some basic properties of FT [6, 30], ST [37, 53], and continuous WT [9]. Only the continuous WT, including its discretized form (which differs from the discrete wavelet transform), is used in the present study. Unless otherwise indicated, WT refers to the continuous WT and its discretized form in the following. The summary provides the basis for the proposed iterative simulation algorithm to be described in the next sections.

Let *x*(*t*) denote a realization of a stochastic process such as the ground motion record, *X*(*t*). FT of *x*(*t*), and its inverse (IFT) can be expressed as

1 $$ \hat{x}(f)={FT}_t\left(x(t)\right)={\int}_{-\infty}^{+\infty }x(t){e}^{-i2\pi ft} dt, $$

and,

2 $$ x(t)={IFT}_f\left(\hat{x}(f)\right)={\int}_{-\infty}^{+\infty}\hat{x}(f){e}^{i2\pi ft} dt\;x(t)={IFT}_f\left(\hat{x}(f)\right)={\int}_{-\infty}^{+\infty}\hat{x}(f){e}^{i2\pi ft} dt $$

where *FT*(•) and *IFT*(•) denote the FT and IFT operations, the subscript associated with these operators indicates the domain or the index where the operation is carried out; $\hat{x}(f)$ denotes FT of *x*(*t*); *f* is the frequency in Hz, $\hat{x}(f)={\hat{x}}^{\ast}\left(-f\right)$, and * denotes the complex conjugate. A symbol or function with a circumflex is used to represent its FT throughout the present study. If *x*(*t*) is given in the discrete form *x*(*j*Δ_*t*_), *j* = 0, …, *N* − 1, with a sampling time interval Δ_*t*_ and the duration *T*, *T* = *N*Δ_*t*_, the (discretized) FT pair is given by,

3 $$ x\left(j{\Delta}_t\right)={IFT}_p\left(\hat{x}\left(p{\Delta}_f\right)\right)=\frac{1}{N{\Delta}_t}\sum \limits_{k=-N/2+1}^{N/2}\hat{x}\left(k{\Delta}_f\right){e}^{i\frac{2\pi }{N} kj}, for\;j=0,\dots, N-1 $$

and,

4 $$ \hat{x}\left(p{\Delta}_f\right)={FT}_j\left(x\left(j{\Delta}_t\right)\right)={\Delta}_t\sum \limits_{k=0}^{N-1}x\left(k{\Delta}_t\right){e}^{-i\frac{2\pi }{N} pk}, for\;p=-N/2+1,\dots, N/2 $$

where Δ_*f*_ = 1/*T*, and the operators *FT*(•) and *IFT*(•) that are used for continuous FT are used for discrete FT as well. It is considered implicitly in the following that the numerical calculations of $\hat{x}\left(p{\Delta}_f\right)$ and *x*(*j*Δ_*t*_) are to be carried by using the fast Fourier transform (FFT) [30] for computational efficiency. Moreover, the notation {•}_*N*_ is used for the collection of its argument of length *N*. For example, {*x*(*j*Δ_*t*_)}_*N*_ represents all *x*(*j*Δ_*t*_) for *j* = 0, …, *N* − 1.

ST of *x*(*t*) is defined as [37, 53],

5 $$ {x}_{\mathbf{\mathcal{S}}}\left(f,\tau \right)= ST\left(x(t)\right)=\underset{-\infty }{\overset{\infty }{\int }}x(t)w\left(f,\tau -t\right){e}^{-i2\pi ft} dt, $$

where ${x}_{\mathbf{\mathcal{S}}}\left(f,\tau \right)$ is the ST coefficient, *ST*(·) denotes the S-transform of its argument, and τ is the center of the window function *w*(*f*, *τ* − *t*) defined as,

6 $$ w\left(f,\tau -t\right)=\frac{\left|f\right|}{\sqrt{2\pi}\kappa}\exp \left(-\frac{f^2{\left(\tau -t\right)}^2}{2{\kappa}^2}\right). $$

The parameter κ in Eq. (6) controls the effective width of the window in ST. It can be shown [53] that,

7 $$ {x}_{\mathbf{\mathcal{S}}}\left(f,\tau \right)=\underset{-\infty }{\overset{\infty }{\int }}\hat{x}\left(\phi +f\right)\exp \left(-\frac{1}{2}{\left(\frac{2\pi \phi \kappa}{f}\right)}^2\right){e}^{i2\pi \phi \tau} d\phi, $$

and,

8 $$ x(t)= IST\left({x}_{\mathbf{\mathcal{S}}}\left(f,\tau \right)\right)=\underset{-\infty }{\overset{\infty }{\int }}\left[\underset{-\infty }{\overset{\infty }{\int }}{x}_{\mathbf{\mathcal{S}}}\left(f,\tau \right) d\tau \right]{e}^{i2\pi ft} df, $$

where *IST*(·) is the inverse S-transform (IST). Using Eqs. (7) and (8), the discretized version of *x*(*t*) and ${x}_{\mathbf{\mathcal{S}}}\left(f,\tau \right)$, represented by *x*(*j*Δ_*t*_) and ${x}_{\mathbf{\mathcal{S}}}\left(q{\Delta}_f,p{\Delta}_t\right)$ pair, can be written as,

9 $$ x\left(j{\Delta}_t\right)= IST\left({x}_{\mathbf{\mathcal{S}}}\left(p{\Delta}_f,q{\Delta}_t\right)\right)={IFT}_p\left({\Delta}_t\sum \limits_{k=0}^{N-1}{x}_{\mathbf{\mathcal{S}}}\left(p{\Delta}_f,k{\Delta}_t\right)\right), for\;j=0,\dots, N-1 $$

and,

10 $$ {x}_{\mathbf{\mathcal{S}}}\left(p{\Delta}_f,q{\Delta}_t\right)= ST\left(x\left(q{\Delta}_t\right)\right)={IFT}_j\left(\hat{x}\left(\left(j+p\right){\Delta}_f\right)\exp \left(-\frac{2{\pi}^2{j}^2{\kappa}^2}{p^2}\right)\right), for\;p=-N/2+1,\dots, N/2, and\;q=0,\dots, N-1 $$

indicating that the evaluation of the ST coefficients at (*p*Δ_*f*_, *q*Δ_*t*_) and its inverse at *j*Δ_*t*_ is based on FT.

WT is defined as [9, 35],

11 $$ {x}_{\mathbf{\mathcal{W}}}\left(s,\tau \right)= WT\left(x(t)\right)=\frac{1}{\sqrt{\left|s\right|}}\underset{-\infty }{\overset{\infty }{\int }}x(t)\psi \ast \left(\frac{t-\tau }{s}\right) dt, $$

where ${x}_{\mathbf{\mathcal{W}}}\left(s,\tau \right)$ is the wavelet coefficient, the operator *WT*(•) denotes WT, *ψ*(⋅) is the mother wavelet and, *s* is the scaling or dilation factor, and τ is the translation or position parameter. Eq. (11) can be expressed as [9, 35],

12 $$ {x}_{\mathbf{\mathcal{W}}}\left(s,\tau \right)=\sqrt{\left|s\right|}\underset{-\infty }{\overset{\infty }{\int }}\hat{x}(f){\hat{\psi}}^{\ast }(sf){e}^{i2\pi f\tau} df, $$

to facilitate its computation by using FFT for signals given in the discretized form. If the admissibility condition 0 < *C*_*ψ*_ < ∞ is satisfied, where ${C}_{\psi }=\underset{-\infty }{\overset{\infty }{\int }}\left(1/\left|f\right|\right)\times {\left|\hat{\psi}(f)\right|}^2 df$, *x*(*t*) can be obtained using the following inverse WT [9],

13 $$ x(t)= IWT\left({x}_{\mathbf{\mathcal{W}}}\left(s,\tau \right)\right)=\frac{1}{C_{\psi }}\underset{-\infty }{\overset{\infty }{\int }}\underset{-\infty }{\overset{\infty }{\int }}{x}_{\mathbf{\mathcal{W}}}\left(s,\tau \right)\frac{1}{\sqrt{\left|s\right|}}\psi \left(\frac{t-\tau }{s}\right)\frac{1}{s^2} d\tau ds, $$

where *IWT*(•) is the inverse of *WT*(•). If *ψ*(*t*) = *ψ* ∗ (−*t*), Eq. (13) becomes,

14 $$ x(t)=\frac{2}{C_{\psi }}\underset{0}{\overset{\infty }{\int }}\underset{-\infty }{\overset{\infty }{\int }}{x}_{\mathbf{\mathcal{W}}}\left(s,\tau \right)\frac{1}{\sqrt{s}}\psi \left(\frac{t-\tau }{s}\right)\frac{1}{s^2} d\tau ds. $$

Moreover, if the analytical wavelet – complex-valued wavelet function that its FT is null for negative frequency – is used, Eq. (13) can be expressed in Morlet formulation [45],

15 $$ x(t)=\operatorname{Re}\left(\frac{2}{C_{1\psi }}\underset{0}{\overset{\infty }{\int }}\frac{1}{s^{3/2}}{x}_{\mathbf{\mathcal{W}}}\left(s,t\right) ds\right), $$

where ${C}_{1\psi }=\underset{0}{\overset{\infty }{\int }}\left({\hat{\psi}}^{\ast }(f)/\left|f\right|\right) df$ and Re() denotes the real part of a complex number.

There are several well-known wavelet families [9, 33, 35], including Daubechies wavelets, generalized Morse wavelets, and Morlet wavelets.

Eqs. (14), (15) and (12) can be written in the following discretized form,

16 $$ x\left(j{\Delta}_t\right)=\frac{2{\Delta}_t\ln {s}_0}{C_{\psi }}\sum \limits_{k=0}^K\sum \limits_{r=-{L}_l}^{L_u}{x}_{\mathbf{\mathcal{W}}}\left({c}_0{s}_0^k,r{\Delta}_t\right)\frac{1}{{\left({c}_0{s}_0^k\right)}^{3/2}}\psi \left(\frac{j{\Delta}_t-r{\Delta}_t}{c_0{s}_0^k}\right), for\;j=0,\dots, N-1 $$

17 $$ x\left(j{\Delta}_t\right)=\operatorname{Re}\left(\frac{2\ln {s}_0}{C_{1\psi }}\sum \limits_{k=0}^K\frac{x_{\mathbf{\mathcal{W}}}\left({c}_0{s}_0^k,j{\Delta}_t\right)}{\sqrt{c_0{s}_0^k}}\right), for\;j=0,\dots, N-1 $$

and,

18 $$ {x}_{\mathbf{\mathcal{W}}}\left({c}_0{s}_0^p,q{\Delta}_t\right)=\sqrt{\left|{c}_0{s}_0^p\right|}\times {IFT}_k\left(\hat{x}\left(k{\Delta}_f\right){\hat{\psi}}^{\ast}\left({c}_0{s}_0^pk{\Delta}_f\right)\right), for\;p=0,\dots, K,\mathrm{and}\ q=0,\dots, N-1 $$

where *c*_0_ and *s*_0_ are parameters for the numerical computation; *K* is the total number of scales considered for the numerical integration; *T*_*L*_ =  − *L*_*l*_Δ_*t*_ and *T*_*U*_ = *L*_*U*_Δ_*t*_ define the lower and upper limits for the integral over time τ, and ${C}_{1\psi }=\underset{0}{\overset{\infty }{\int }}\left({\hat{\psi}}^{\ast }(f)/\left|f\right|\right) df$. In the following, we restrict ourselves to the real-valued signal and the analytical wavelets or wavelets with *ψ*(*t*) = *ψ*^∗^(−*t*).

## Gaussian process, power spectral density, and defining process in the transform domain

According to the spectral representation method [47] with the use of FFT [59], a sample of a Gaussian stationary process, *x*(*t*), can be simulated by transforming Gaussian white noise *w*(*t*) to the Fourier domain, multiplying it with an intensity function $\left|\hat{y}(f)\right|$, and transforming it back to the time domain. That is,

19 $$ x(t)={IFT}_f\left(\left|y(f)\right|{e}^{i{\theta}_{\mathbf{\mathcal{F}}}\left(w(t)\right)}\right), $$

where ${e}^{i{\theta}_{\mathbf{\mathcal{F}}}\left(w(t)\right)}=\eta \left( FT\left(w(t)\right)\right)$, in which the function *η*(*C*) = *C*/|*C*| is introduced to normalize the complex number *C*. Based on FT pair, $\hat{x}(f)=\left|y(f)\right|{e}^{i{\theta}_{\mathbf{\mathcal{F}}}\left(w(t)\right)}$. Since, by definition, the double-sided PSD function of the process *x*(*t*) with duration *T*, ${S}_{\mathbf{\mathcal{F}}x}(f)$, is given by,

20 $$ {S}_{\mathbf{\mathcal{F}}x}(f)=\hat{x}(f){\hat{x}}^{\ast }(f)/T, $$

it indicates that given the target PSD function ${S}_{\mathbf{\mathcal{F}}x}(f)$, one could define a stationary Gaussian process in the Fourier domain by assigning $\left|\hat{y}(f)\right|=\left|\hat{x}(f)\right|=\sqrt{S_{\mathbf{\mathcal{F}}x}(f)T}$. The samples of the process so defined can be obtained using,

21 $$ x(t)={IFT}_f\left(\left|\hat{x}(f)\right|{e}^{i{\theta}_{\mathbf{\mathcal{F}}}\left(w(t)\right)}\right), $$

and the expected PSD of the sampled signals equals the prescribed ${S}_{\mathbf{\mathcal{F}}x}(f)$. The use of the definition given in Eq. (20) preserves the energy of *x*(*t*) according to Parseval’s theory.

We note that by assigning $\left|\hat{y}(f)\right|$ equal to $M(t)\left|\hat{x}(f)\right|$, Eq. (19) becomes,

22 $$ x(t)={IFT}_f\left(M(t)\times \left|\hat{x}(f)\right|{e}^{i{\theta}_{\mathbf{\mathcal{F}}}\left(w(t)\right)}\right)=M(t)\times {IFT}_f\left(\left|\hat{x}(f)\right|{e}^{i{\theta}_{\mathbf{\mathcal{F}}}\left(w(t)\right)}\right), $$

which simulates a uniformly amplitude modulated evolutionary process [38]. Such a process has an evolutionary PSD function equals ${\left|M(t)\right|}^2\left(\hat{x}(f){\hat{x}}^{\ast }(f)/T\right)$, and *M*(*t*) is the amplitude modulation function, which will be considered to be positive. However, the use of |*y*(*f*)| equal to $M\left(t,f\right)\left|\hat{x}(f)\right|$ in Eq. (19) does not lend itself to be interpreted as a proper inverse Fourier transform because the modulation function depends on the frequency. This reduces the computational efficiency that otherwise can be gained by using FFT; it also makes the distinction between the modulation function and intensity function more blurred. We will concentrate only on the case where the modulation function is defined outside of the transform domain. However, the consideration of modulation that depends on variables in the transform domain could be a valid assumption.

Maraun et al. [26] emphasized the usefulness of using Eq. (19) to obtain samples of stationary Gaussian process, and extended it to define a class of nonstationary Gaussian processes in the wavelet domain by the wavelet multipliers $\left|{y}_{\mathbf{\mathcal{W}}}\left(s,\tau \right)\right|$, indicating that an individual process is defined by its multipliers and a synthesizing wavelet pair. Samples of *x*(*t*) based on such a definition are then given as,

23 $$ x(t)= IWT\left(\left|{y}_{\mathbf{\mathcal{W}}}\left(s,\tau \right)\right|{e}^{i{\theta}_{\mathbf{\mathcal{W}}}\left(w(t)\right)}\right), $$

where ${e}^{i{\theta}_{\mathbf{\mathcal{W}}}\left(w(t)\right)}=\eta \left( WT\left(w(t)\right)\right)$. We use the intensity function $\left|{y}_{\mathbf{\mathcal{W}}}\left(s,\tau \right)\right|$ and ${e}^{i{\theta}_{\mathbf{\mathcal{W}}}\left(w(t)\right)}$ in Eq. (23) instead of using ${y}_{\mathbf{\mathcal{W}}}\left(s,\tau \right)$ and *WT*(*w*(*t*)) as suggested in Maraun et al. [26]. The use of ${e}^{i{\theta}_{\mathbf{\mathcal{W}}}\left(w(t)\right)}$ instead of *WT*(*w*(*t*)) is aimed at not biasing the energy arising from the intensity function since [*WT*(*w*(*t*))][*WT*(*w*(*t*))]^∗^ is not a constant in the wavelet domain by using WT defined in Eq. (12). The use of $\left|{y}_{\mathbf{\mathcal{W}}}\left(s,\tau \right)\right|$ (as well as $\left|\hat{y}(f)\right|$ in Eqs. (21) and (22)) is more restrictive than ${y}_{\mathbf{\mathcal{W}}}\left(s,\tau \right)$ but is adequate for the proposed algorithm in the following section since we are focused on real-valued signals. However, a negatively valued intensity and complex-valued intensity may be considered for other applications.

Similar to the use of *M*(*t*) in defining the uniformly modulated evolutionary process mentioned earlier, we include *M*(*t*) in Eq. (23),

24 $$ x(t)= IWT\left(M(t)\left|{y}_{\mathbf{\mathcal{W}}}\left(s,\tau \right)\right|{e}^{i{\theta}_{\mathbf{\mathcal{W}}}\left(w(t)\right)}\right)=M(t)\times IWT\left(\left|{y}_{\mathbf{\mathcal{W}}}\left(s,\tau \right)\right|{e}^{i{\theta}_{\mathbf{\mathcal{W}}}\left(w(t)\right)}\right), $$

to define a **mod**ulated and **i**ntensity **f**unction adjusted (MODIF) process. The intensity function gives time-scale characteristics of the process, and the modulation function provides additional time-varying characteristics of the process.

We further extend the concept of defining the MODIF process in the time-frequency domain according to ST, denoted as the S-domain, where samples of *x*(*t*) are given as,

25 $$ x(t)= IST\left(M(t)\times \left|{y}_{\mathbf{\mathcal{S}}}\left(f,\tau \right)\right|{e}^{i{\theta}_{\mathbf{\mathcal{S}}}\left(w(t)\right)}\right)=M(t)\times IST\left(\left|{y}_{\mathbf{\mathcal{S}}}\left(f,\tau \right)\right|{e}^{i{\theta}_{\mathbf{\mathcal{S}}}\left(w(t)\right)}\right), $$

where ${y}_{\mathbf{\mathcal{S}}}\left(f,\tau \right)$ is an intensity function in the S-domain, and ${e}^{i{\theta}_{\mathbf{\mathcal{S}}}\left(w(t)\right)}=\eta \left( ST\left(w(t)\right)\right)$.

It is noted that besides the above-mentioned transforms, there are other transforms used for signal analysis and modeling; for example, the generalized Fourier family transforms [2]. Therefore, it is relevant and straightforward to conceptually generalize the approach in defining the MODIF processes in the transform domain if other transform pair is considered. The definitions lend themselves to an easily understandable and almost trivial algorithm to simulate stochastic processes:

A) Sample Gaussian white noise, *w*(*t*), and calculate the normalized coefficients of *w*(*t*) in the transform domain (e.g., ${e}^{i{\theta}_{\mathbf{\mathcal{F}}}\left(w(t)\right)}$, or ${e}^{i{\theta}_{\mathbf{\mathcal{W}}}\left(w(t)\right)}$, or ${e}^{i{\theta}_{\mathbf{\mathcal{S}}}\left(w(t)\right)}$ if FT, or WT, or ST is used, respectively).
B) Apply the inverse transform to the product of the intensity function and the normalized coefficients obtained in Step A).
C) Apply the modulation function to the simulated signal from Step B).

Step C) is separated from Steps A) and B) and is not affected by the selected transformation. A critical issue of applying the MODIF process with prescribed target energy distribution is that the energy distribution of the sampled signals for given intensity function may not be readily established, except for the case where FT is used (i.e., transforms with non-redundant representation). This is because unlike the FT, both WT and ST provide redundant representation. The redundant representation results in that, in general, $\left|{y}_{\mathbf{\mathcal{W}}}\left(s,\tau \right)\right|{e}^{i{\theta}_{\mathbf{\mathcal{W}}}\left(w(t)\right)}$ and $\left|{y}_{\mathbf{\mathcal{S}}}\left(s,\tau \right)\right|{e}^{i{\theta}_{\mathbf{\mathcal{S}}}\left(w(t)\right)}$ do not represent the proper coefficients of WT and ST, respectively. In other words, $\left|{y}_{\mathbf{\mathcal{W}}}\left(s,\tau \right)\right|{e}^{i{\theta}_{\mathbf{\mathcal{W}}}\left(w(t)\right)}$ and $\left|{y}_{\mathbf{\mathcal{S}}}\left(s,\tau \right)\right|{e}^{i{\theta}_{\mathbf{\mathcal{S}}}\left(w(t)\right)}$ are not equal to ${x}_{\mathbf{\mathcal{W}}}\left(s,\tau \right)= WT\left( IWT\left(\left|{y}_{\mathbf{\mathcal{W}}}\left(s,\tau \right)\right|{e}^{i{\theta}_{\mathbf{\mathcal{W}}}\left(w(t)\right)}\right)\right)$ and ${x}_{\mathbf{\mathcal{S}}}\left(f,\tau \right)= ST\left( IST\left(\left|{y}_{\mathbf{\mathcal{S}}}\left(s,\tau \right)\right|{e}^{i{\theta}_{\mathbf{\mathcal{S}}}\left(w(t)\right)}\right)\right)$, respectively.

To see the impact of this inequality on the simulated MODIF process by using Eq. (24), we note that we can define the double-sided time-scale PSD (TSPSD) function of the simulated process *x*(*t*), ${S}_{\mathbf{\mathcal{W}}x}\left(s,\tau \right)$, as,

26 $$ {S}_{\mathbf{\mathcal{W}}x}\left(s,\tau \right)=\left(\frac{x_{\mathbf{\mathcal{W}}}\left(s,\tau \right)}{s\sqrt{C_{\psi }}}\right){\left(\frac{x_{\mathbf{\mathcal{W}}}\left(s,\tau \right)}{s\sqrt{C_{\psi }}}\right)}^{\ast }. $$

The use of this definition leads to energy preservation since the integral of ${S}_{\mathbf{\mathcal{W}}x}\left(s,\tau \right)$ in the wavelet domain equals the integral of |*x*(*t*)|^2^ in the time domain (see Proposition 2.4.1 in Daubechies [9]). Consequently, even we assign $\left|{y}_{\mathbf{\mathcal{W}}}\left(s,\tau \right)\right|$ equals $\sqrt{S_{\mathbf{\mathcal{W}}x}\left(s,\tau \right){C}_{\psi }}\left|s\right|$ and *M*(*t*) = 1 for the simulation, the average energy of the sampled signals according to Eq. (24) will likely deviate from the specified target ${S}_{\mathbf{\mathcal{W}}x}\left(s,\tau \right)$.

Consider that we simulate the MODIF process using Eq. (25). We can define the double-sided time-frequency PSD (TFPSD) function of the simulated process, ${S}_{\mathbf{\mathcal{S}}x}\left(f,\tau \right)$, as,

27 $$ {S}_{\mathbf{\mathcal{S}}x}\left(f,\tau \right)=\left({x}_{\mathbf{\mathcal{S}}}\left(f,\tau \right)/\sqrt{D_{\kappa}\left|f\right|}\right){\left({x}_{\mathbf{\mathcal{S}}}\left(f,\tau \right)/\sqrt{D_{\kappa}\left|f\right|}\right)}^{\ast }, $$

since the use of this definition leads to energy preservation [18], where ${D}_{\kappa }=\underset{-\infty }{\overset{\infty }{\int }}\left(1/\left|\zeta \right|\right)\times \exp \left(-{\left(2\pi \kappa \left(\zeta -1\right)\right)}^2\right) d\zeta$. However, the average energy of the sampled signals by using Eq. (25) with $\left|{y}_{\mathbf{\mathcal{S}}}\left(f,\tau \right)\right|$ equal to $\left|{x}_{\mathbf{\mathcal{S}}}\left(f,\tau \right)\right|=\sqrt{S_{\mathbf{\mathcal{S}}x}\left(f,\tau \right){D}_{\kappa}\left|f\right|}$ and *M*(*t*) = 1 will likely deviate from the specified target.

In addition to the discussed energy distortion, the application of the MODIF process is likely to lead to the samples obtained from Eqs. (22), (24) and (25) to follow a marginal cumulative distribution function (CDF) that deviates from the prescribed marginal CDF of the zero-mean process *F*_*X*, *t*_(*x*(*t*)). An iterative process is proposed in the following sections to simulate the nonstationary and non-Gaussian with prescribed target PSD and CDF. The PSD functions that satisfy the energy preservation by considering the selected transform are used as the basis to describe the proposed algorithm to maintain consistency. Although this could become clumsy in some instances, it is useful in checking that a consistent transform pair is employed.

## Iterative power and amplitude correction algorithm

### IAAFT algorithm

To develop the proposed iterative algorithm, we note that, given the observed {*x*(*j*Δ_*t*_)}_*N*_, the IAAFT algorithm was proposed by Schreiber and Schmitz [43, 44] in the context of generating surrogates for statistical hypothesis testing. The algorithm repeatedly uses FT and IFT, and ranked data. This algorithm is explained using the ranking of *x*(*j*Δ_*t*_) in the following.

The PSD function ${S}_{\mathbf{\mathcal{F}}x}(f)$ of {*x*(*j*Δ_*t*_)}_*N*_ is calculated using Eq. (19) with possible smoothing. The objective of IAFFT is to generate surrogates that match the calculated ${S}_{\mathbf{\mathcal{F}}x}(f)$ and shuffled {*x*(*j*Δ_*t*_)}_*N*_. A similar algorithm - the spectral correction algorithm - was independently designed by Masters and Gurley [27] to simulate non-Gaussian processes for the given target ${S}_{\mathbf{\mathcal{F}}x}(f)$ and target marginal CDF *F*_*X*_(*x*(*t*)). A subtle difference between these two algorithms is how the prescribed target PSD function and CDF are obtained or assigned. For example, {*x*(*j*Δ_*t*_)}_*N*_ is obtained through distribution mapping in the spectral correction algorithm. In IAAFT, {*x*(*j*Δ_*t*_)}_*N*_ is given and shuffled. This shuffling, in the spectral correction method, can be viewed as matching the prescribed probability distribution. Once {*x*(*j*Δ_*t*_)}_*N*_ is prescribed and ${S}_{\mathbf{\mathcal{F}}x}(f)$ is calculated, by letting {*ξ*(*j*)}_*N*_ equal to the ascendingly sorted {*x*(*j*Δ_*t*_)}_*N*_, the steps of the IAAFT algorithm are:

1. Sample a sequence of Gaussian white noise, *w*(*t*), of length *N*, calculate ${e}^{i{\phi}_p}=\eta \left( FT\left(w(t)\right)\right)$;
2. Calculate ${x}_{PC}\left(j{\Delta}_t\right)={IFT}_p\left(\left|\sqrt{S_{\mathbf{\mathcal{F}}x}\left(p{\Delta}_f\right)T}\right|{e}^{i{\phi}_p}\right)$ and find the ranking of *x*_*PC*_(*j*Δ_*t*_), *r*_*j*_, for *j* = 0, …, *N* − 1, based on the ascending order;
3. Set *x*_*AC*_(*j*Δ_*t*_) = *ξ*(*r*_*j*_), for *j* = 0, …, *N* − 1; and calculate ${e}^{i{\phi}_p}=\eta \left({FT}_j\left({x}_{AC}\left(j{\Delta}_t\right)\right)\right)$, and.
4. Repeat Steps 2) to 3) until the convergence criterion is achieved.

Steps 1) and 2) are the same as Steps A) and B) described earlier that simulates a Gaussian process, except an additional ranking of *x*_*PC*_(*j*Δ_*t*_) is carried out, which is equivalent to define the CDF as a preparation for the iteration. In general, Step 2) leads to *x*_*PC*_(*j*Δ_*t*_) with the PSD correction but may deviate from the target CDF assigned by {*ξ*(*j*)}_*N*_, and Step 3) leads to the sampled *x*_*AC*_(*j*Δ_*t*_) with the amplitude correction (i.e., matching CDF assigned based on {*ξ*(*j*)}_*N*_) but may deviate from the target PSD. The iteration adjusts the PSD and CDF of the sampled time series to their corresponding targets. The tolerable differences between *x*_*PC*_(*j*Δ_*t*_) and *x*_*AC*_(*j*Δ_*t*_) can be used as the convergence criterion. Once convergence is achieved *x*_*PC*_(*j*Δ_*t*_) or *x*_*AC*_(*j*Δ_*t*_) can be used as the sampled time series. Note that since the time increment equals Δ_*t*_, the corresponding Nyquist frequency for the considered signal equals 1/(2Δ_*t*_).

The IAAFT algorithm is designed for stationary processes. For the shuffling of {*x*(*j*Δ_*t*_)}_*N*_ to simulation stationary process, it is implicitly considered that the marginal CDF of *x*(*t*) at any given time remains to be the same. Also, the PSD function for the stationary process is time-independent. The IAAFT algorithm or the spectral correction method is not applicable to simulate nonstationary processes as they have time-varying PSD and CDF.

#### Proposed iterative power and amplitude correction algorithm

In this section, we describe the proposed iterative power and amplitude correction (IPAC) algorithm to simulate the time history {*x*(*j*Δ_*t*_)}_*N*_ of a zero-mean nonstationary non-Gaussian process. Since Δ_*t*_ is assigned, *N* can be determined based on the length of the signal to be simulated and vice versa, and the Nyquist frequency for the sampled signal equals 1/(2Δ_*t*_). The proposed algorithm could be viewed as an extension to the IAAFT algorithm. For the simulation, it is considered that, for *M*(*t*) = 1, the PSD function of the process that is characterized based on FT, or ST, or WT is given, and the distribution type for the marginal CDF of *x*(*t*), *F*_*X*, *t*_(*x*(*t*)), is known. Moreover, it is considered that *F*_*X*, *t*_(*x*(*t*)) can be completely defined by the zero-mean, the time-varying standard deviation, σ(*t*), and other prescribed distribution parameters if they are required (since, in some cases, a CDF with more than two parameters may be considered).

If FT is considered for a stationary process, the standard deviation σ(*t*) equals $\sqrt{\lambda_{\mathbf{\mathcal{F}}x}}$ which is time-independent, where ${\lambda}_{\mathbf{\mathcal{F}}x}$, equals the integral of ${S}_{\mathbf{\mathcal{F}}x}(f)$ over the frequency domain. Since ${S}_{\mathbf{\mathcal{S}}x}\left(f,\tau \right)$ provides the energy distribution over the time-frequency domain, the integral of ${S}_{\mathbf{\mathcal{S}}x}\left(f,\tau \right)$ over the frequency domain provides the energy distribution in the time domain, ${\lambda}_{\mathbf{\mathcal{S}}x}\left(\tau \right)$,

28 $$ {\lambda}_{\mathbf{\mathcal{S}}x}\left(\tau \right)=\underset{-\infty }{\overset{\infty }{\int }}{S}_{\mathbf{\mathcal{S}}x}\left(f,\tau \right) df, $$

and the integral of ${S}_{\mathbf{\mathcal{S}}x}\left(f,\tau \right)$ over the time domain provides the energy distribution in the frequency domain, ${S}_{\mathbf{\mathcal{S}}x}(f)$. Analogously to the statistics for the stationary process, ${\lambda}_{\mathbf{\mathcal{S}}x}\left(\tau \right)$ represents the variance of *x*(τ), and σ(*t*) equals $\sqrt{\lambda_{\mathbf{\mathcal{S}}x}(t)}$. Similarly, for the given ${S}_{\mathbf{\mathcal{W}}x}\left(s,\tau \right)$, the time-varying variance ${\lambda}_{\mathbf{\mathcal{W}}x}\left(\tau \right)$ is given by,

29 $$ {\lambda}_{\mathbf{\mathcal{W}}x}\left(\tau \right)=\underset{-\infty }{\overset{\infty }{\int }}{S}_{\mathbf{\mathcal{W}}x}\left(s,\tau \right) df, $$

and σ(*t*) equals $\sqrt{\lambda_{\mathbf{\mathcal{W}}x}(t)}$. The integral of ${S}_{\mathbf{\mathcal{W}}x}\left(s,\tau \right)$ over the time domain provides the energy distribution in the scale domain, ${S}_{\mathbf{\mathcal{W}}x}(s)$.

**Fig. 1:**
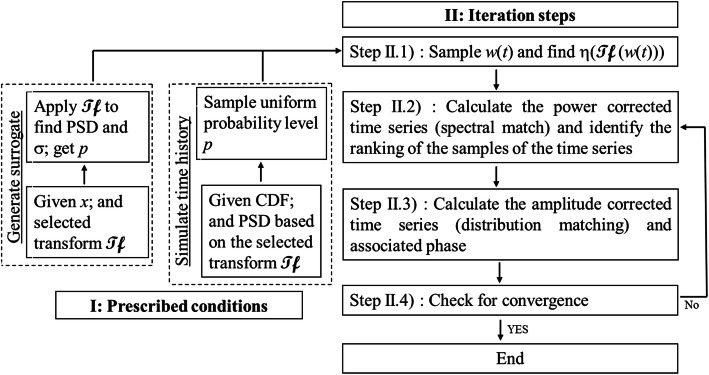
Iterative power and amplitude correction algorithm to simulate nonstatinary and non-Gaussian processes (Tf denotes the selected transform in this figure)

Let *u*(*t*) be a uniformly distributed random variable between 0 and 1 with its marginal CDF denoted as *U*(*u*(*t*)). The relation between *u*(*t*) and *x*(*t*) can be established based on the probability transformation, *U*(*u*(*t*)) = *F*_*X*, *t*_(*x*(*t*)). The steps in the IPAC algorithm in a pseudo-code form are shown in the flowchart depicted in Fig. 1 and are described as follows:

I) Prescribe the targets and initiate the simulation process:

Sample {*u*(*j*Δ_*t*_)}_*N*_ based on a random number generation algorithm for a uniformly distributed random variable between 0 and 1. Assign {*p*(*j*)}_*N*_ equal to the ascendingly sorted {*u*(*j*Δ_*t*_)}_*N*_, and the intensity function $\left|{y}_{\mathbf{\mathcal{Tf}}}\left({\overset{\rightharpoonup }{s}}_p\right)\right|$ according to the considered transform pair (*Tf*(•), *ITf*(•)), where.

- For (*Tf*(•), *ITf*(•)) = (*FT*(•), *IFT*(•)), $\left|{y}_{\mathbf{\mathcal{Tf}}}\left({\overset{\rightharpoonup }{s}}_p\right)\right|=\left|{y}_{\mathbf{\mathcal{F}}}(f)\right|=\sqrt{S_{\mathbf{\mathcal{F}}x}(f)T}$, and $\sigma (t)=\sqrt{\lambda_{\mathbf{\mathcal{F}}x}}$ which is time-independent,
- For (*Tf*(•), *ITf*(•)) = (*ST*(•), *IST*(•)), $\left|{y}_{\mathbf{\mathcal{Tf}}}\left({\overset{\rightharpoonup }{s}}_p\right)\right|=\left|{y}_{\mathbf{\mathcal{S}}}\left(f,\tau \right)\right|=\sqrt{S_{\mathbf{\mathcal{S}}x}\left(f,\tau \right){D}_{\kappa}\left|f\right|}$, and $\sigma (t)=\sqrt{\lambda_{\mathbf{\mathcal{S}}x}(t)}$,
- For (*Tf*(•), *ITf*(•)) = (*WT*(•), *IWT*(•)), $\left|{y}_{\mathbf{\mathcal{Tf}}}\left({\overset{\rightharpoonup }{s}}_p\right)\right|=\left|{y}_{\mathbf{\mathcal{W}}}\left(s,\tau \right)\right|=\sqrt{S_{\mathbf{\mathcal{W}}x}\left(s,\tau \right){C}_{\psi }}\left|s\right|$, and $\sigma (t)=\sqrt{\lambda_{\mathbf{\mathcal{W}}x}(t)}$.

II) Iteration steps:

II.1) Sample a sequence of Gaussian white noise, *w*(*t*), of length *N*, calculate ${e}^{i{\phi}_{\mathbf{\mathcal{Tf}}}\left({\overset{\rightharpoonup }{s}}_p\right)}=\eta \left( Tf\left(w(t)\right)\right)$;

II.2) Calculate ${x}_{PC}\left(j{\Delta}_t\right)= ITf\left(\left|{y}_{\mathbf{\mathcal{Tf}}}\left({\overset{\rightharpoonup }{s}}_p\right)\right|{e}^{i{\phi}_{\mathbf{\mathcal{Tf}}}\left({\overset{\rightharpoonup }{s}}_p\right)}\right)$, ${p}_{PC}\left(j{\Delta}_t\right)={F}_{X,j{\Delta}_t}\left({x}_{PC}\left(j{\Delta}_t\right)\right)$, and find the rank of *p*_*PC*_(*j*Δ_*t*_), denoted as *r*_*j*_, for *j* = 0, …, *N* − 1;

II.3) Set ${x}_{AC}\left(j{\Delta}_t\right)={F}_{X,j{\Delta}_t}^{-1}\left(p\left({r}_j\right)\right)$, for *j* = 0, …, *N* − 1; and calculate ${e}^{i{\phi}_{\mathbf{\mathcal{Tf}}}\left({\overset{\rightharpoonup }{s}}_p\right)}=\eta \left( Tf\left({x}_{AC}\left(j{\Delta}_t\right)\right)\right)$;

II.4) Repeat Steps II.2) to II.3) until the convergence criterion is satisfied.

II.5) *x*(*j*Δ_*t*_) = *M*(*j*Δ_*t*_) × *x*_*AC*_(*j*Δ_*t*_).

The algorithm essentially simulates the MODIF process and iteratively corrects the PSD and CDF. The intensity function and the transform pair are used from Steps I) to II.4), while the modulation function only affects the assignment of the final results in Step II.5). Since *x*_*AC*_(*j*Δ_*t*_) is used in Step II.5), the distribution match (i.e., matching samples of *F*_*X*_(*X*(*t*)), {*x*(*j*Δ_*t*_)}_*N*_) is ensured by design. One could replace Step II.5) with *x*(*j*Δ_*t*_) = *M*(*j*Δ_*t*_) × *x*_*PC*_(*j*Δ_*t*_) without altering the results if a stringent convergence criterion is employed. As can be observed from the steps of the IPAC algorithm, the analysis, as well as the simulation, is carried out within the same transform pair. It avoids the need to map the obtained results from one type of transform into a different kind of transform (e.g., obtaining the spectrum using continuous WT and then transform it into evolutionary PSD). The algorithm emphasizes the ranking and matching of the probability values. Note that it may be attempting to replace the uniform distribution with the normal distribution for *u*(*t*). However, by doing so, it requires the use of the inverse distribution transformation in Steps II.2 and II.3) and increases computing demand.

The algorithm can be simplified if *F*_*X*, *t*_(*x*(*t*)) remains unchanged and only depends on σ(*t*), that is, the marginal probability distribution of *z*(*t*) = *x*(*t*)/*σ*(*t*), *F*_*Z*_(*z*(*t*)), is time-independent and *z*(*t*) has zero mean and unit variance. In such a case, we calculate ${\left\{\zeta (j)\right\}}_N={\left\{{F}_Z^{-1}\left(p(j)\right)\right\}}_N$ in Step I); we replace “ ${p}_{PC}\left(j{\Delta}_t\right)={F}_{X,j{\Delta}_t}\left({x}_{PC}\left(j{\Delta}_t\right)\right)$ ” in Step II.2) and “ ${x}_{AC}\left(j{\Delta}_t\right)={F}_{X,j{\Delta}_t}^{-1}\left(p\left({r}_j\right)\right)$ ” in Step II.3) with “ *p*_*PC*_(*j*Δ_*t*_) = *x*_*PC*_(*j*Δ_*t*_)/*σ*(*j*Δ_*t*_) ” and “ *x*_*AC*_(*j*Δ_*t*_) = *ζ*(*r*_*j*_)*σ*(*j*Δ_*t*_) ”, respectively. This avoids the use of probability distribution function during the iteration to gain extra computational efficiency. This simplified version can also be used to generate surrogate for observed {*x*(*j*Δ_*t*_)}_*N*_, which has the effect of the modulation function already removed. This is done by calculating {*z*(*j*Δ_*t*_)}_*N*_ = {*x*(*j*Δ_*t*_)/*σ*(*j*Δ_*t*_)}_*N*_, and letting {*ζ*(*j*)}_*N*_ equal to the ascendingly sorted {*z*(*j*Δ_*t*_)}_*N*_ in Step I.1) (instead of ${\left\{\zeta (j)\right\}}_N={\left\{{F}_Z^{-1}\left(p(j)\right)\right\}}_N$), where *σ*(*j*Δ_*t*_) is to be calculated based on the PSD function estimated from {*x*(*j*Δ_*t*_)}_*N*_ by using a preferred transform.

The usefulness of surrogate in the context of wind engineering was presented in McCullough and Kareem [28]. The proposed algorithm, when used with WT to generate surrogate, differs from that given in Chavez and Cazelles [4] for testing time-localized coherence, in that the time-varying *σ*(*j*Δ_*t*_) is neglected in their algorithm (i.e., the amplitude adjustment is based on {*x*(*j*Δ_*t*_)}_*N*_ rather than its normalized version in the IPAC algorithm). This is convenient and may likely speed up the convergence of the algorithm. However, the basis for the shuffling of {*x*(*j*Δ_*t*_)}_*N*_ is unclear if the marginal probability distribution of *x*(*j*Δ_*t*_) for a nonstationary process is assumed to be time-varying.

## Numerical examples

In this section, we illustrate the proposed algorithm by generating surrogate for a given ground motion record and for a given fluctuating component of wind velocity time history of a high-intensity wind event. We apply the algorithm to sample nonstatinary ground motions for prescribed target PSD, where the target is defined based on a set of ground motion records, and the CDF is assumed to be Gaussian and non-Gaussian. The test of the proposed algorithm for esoteric mathematical models is beyond the consideration of the present study.

### Generating surrogate for an earthquake record

**Fig. 2:**
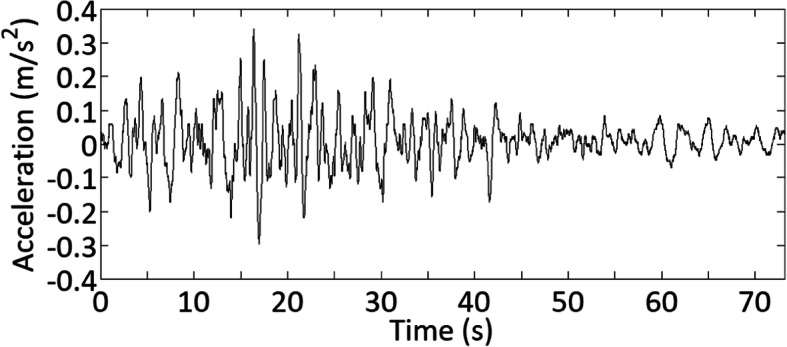
Ground motions recorded at the CU station, UNAM, Mexico, for the Michoacán earthquake that occurred on September 19, 1985

**Fig. 3:**
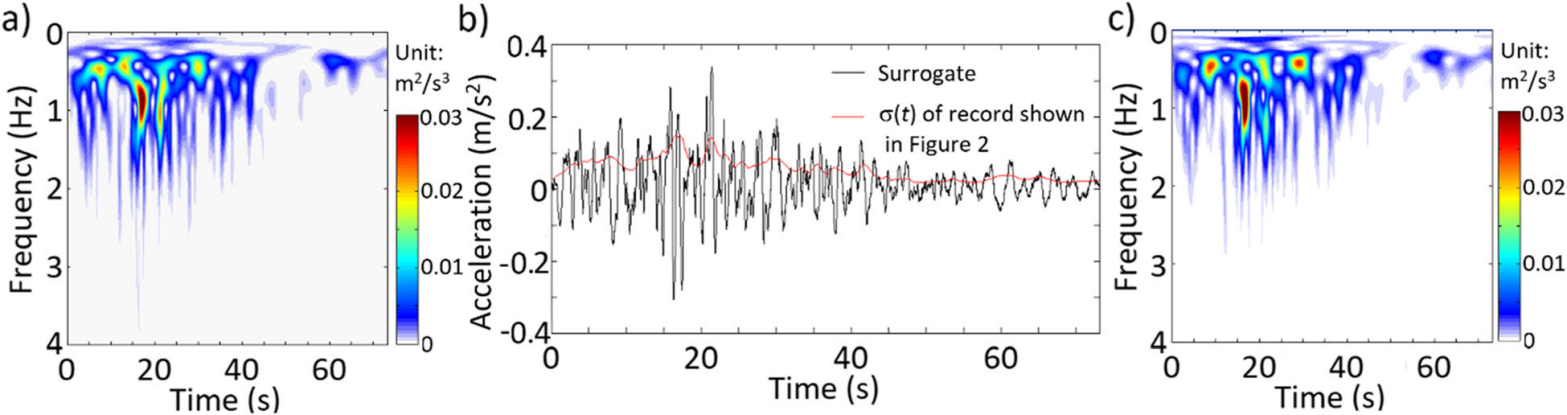
Results by using ST for the given record shown in Fig. 2: **a** TFPSD of the given record, **b** a generated surrogate and σ(*t*) of the given record, and **c** TFPSD of the generated surrogate shown in Fig. 3b

Consider the record shown in Fig. 2. By applying ST with the window parameter κ = 1 (see Eq. (6)), the obtained TFPSD function is shown in Fig. 3a, and the calculated time-varying σ(*t*) is presented in Fig. 3b, showing that the TFPSD varies in time and frequency.

By applying the IPAC algorithm, a surrogate is simulated and shown in Fig. 3b. The TFPSD of this surrogate is depicted in Fig. 3c. The figure shows that the surrogate resembles the given record, and its TFPSD function resembles well that shown in Fig. 3a. As *x*_*AC*_(*t*) is used for generating surrogate (see Step II.5 in IPAC algorithm), the amplitude (or probability distribution) matching is certain, so no plot is provided. Additional test runs indicate that the convergence is usually achieved within five iterations, depending on the adopted convergence criterion. It was noted that the average TFPSD function from multiple generated surrogates tends to be smoother as compared to the TFPSD of the observed record, which is expected since the observed as well as a single sampled record are realizations of stochastic processes.

**Fig. 4:**
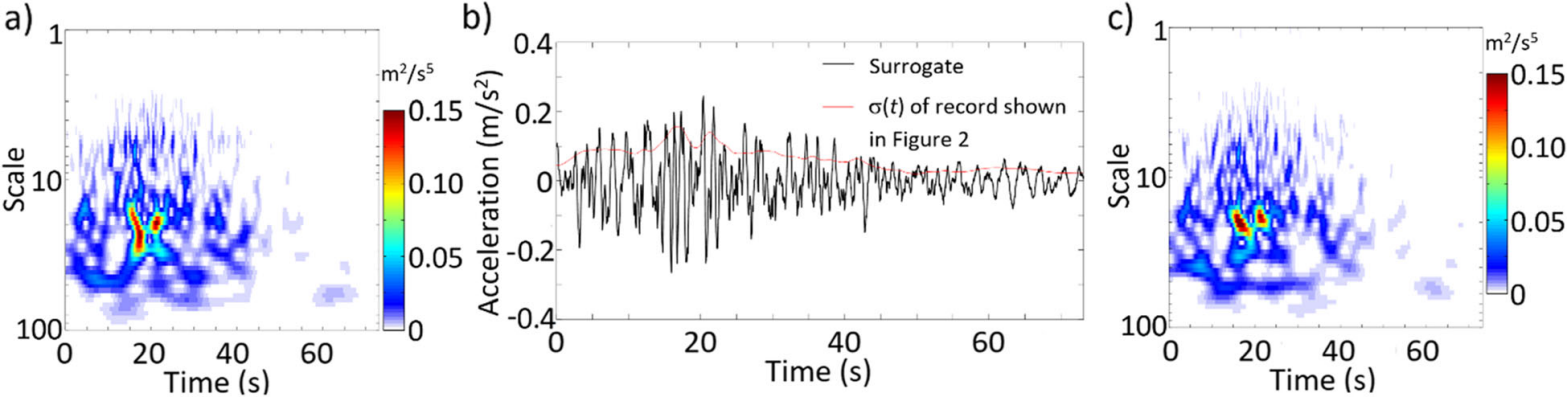
Results by using WT for the given record shown in Fig. 2: **a** TSPSD of the given record, **b** a generated surrogate and σ(*t*) of the given record, and **c** TSPSD of the generated surrogate shown in Fig. 3b

The analysis based on ST is repeated but by applying WT using the generalized morse wavelets (GMWs) [33],

30 $$ {\hat{\psi}}_{0,\beta, \gamma }(f)=U(f){a}_{\beta, \gamma }{\left(2\pi f\right)}^{\beta }{e}^{-{\left(2\pi f\right)}^{\gamma }}, $$

where *U*(*ω*) is the Heaviside function, *a*_*β*, *γ*_ = 2(*eγ*/*β*)^*β*/*γ*^, and β and γ are model parameters. For GMW, *C*_*ψ*_ = 2*a*_2*β*, *γ*_Γ(2*β*, *γ*)/(2*γ*) and *C*_1*ψ*_ = *a*_*β*, *γ*_Γ(*β*, *γ*)/(*γ*). The GMW is an analytical wavelet, and it was used to evaluate the coherence of the seismic ground motions [39]. For the numerical analysis, β = 3, γ = 20, *c*_0_ = 0.528, *s*_0_ = 2^0.1^ and *K* = 91 (see Eq.(17)) are employed since these values are suggested as the default values for the algorithm implemented in MATLAB (Version 2019a). The obtained TSPSD and σ(*t*) of the record are shown in Fig. 4a and b, respectively. A generated surrogate is also shown in Fig. 4b with its corresponding TSPSD function depicted in Fig. 4c. An inspection of the surrogate depicted in Fig. 4b and the original record presented in Fig. 2 indicates that they exhibit similar temporal trends. The TSPSD of the surrogate resembles that of the given record. Again, the convergence is achieved within a few iterations. A comparison of σ(*t*) shown in Figs. 3 and 4b indicates that they are almost identical. The minor differences between them are due to that ST and WT have different time-frequency (or time-scale) decomposition.

#### Generating surrogate for a wind record

**Fig. 5:**
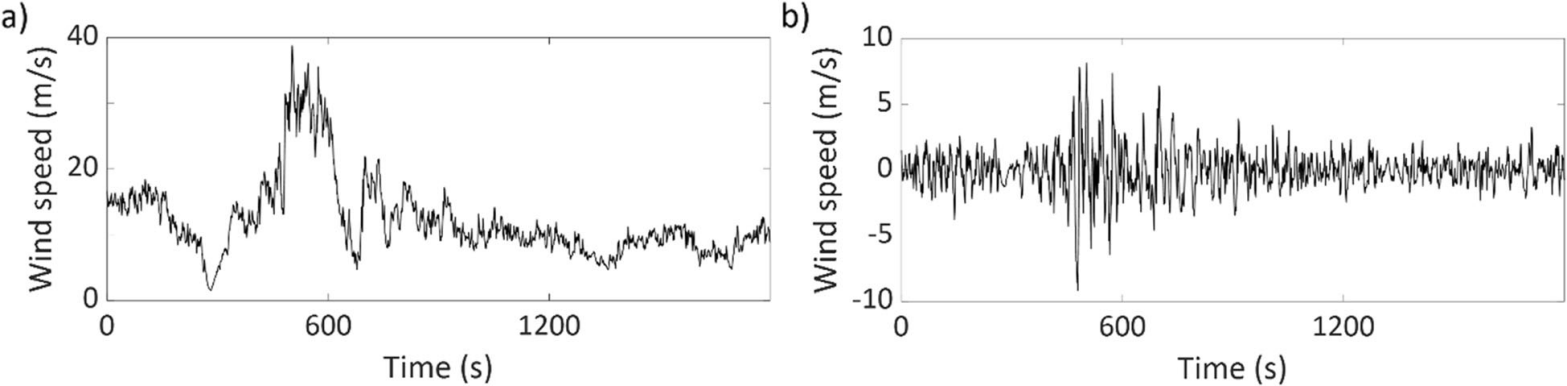
Wind velocity record from Tower 4 and 10 m height of the rear-flank downdraft that occurred during the evening on June 4, 2002, near Lubbock, Texas [13, 34]: **a** the wind record, and **b** the fluctuating component of the record

Now, consider a wind record presented in Fig. 5a. For simplicity, the box window with a width of 32 samples is used to find the mean wind velocity of the time-varying wind record. By removing the mean, the fluctuating component of the wind is presented in Fig. 5b.

**Fig. 6:**
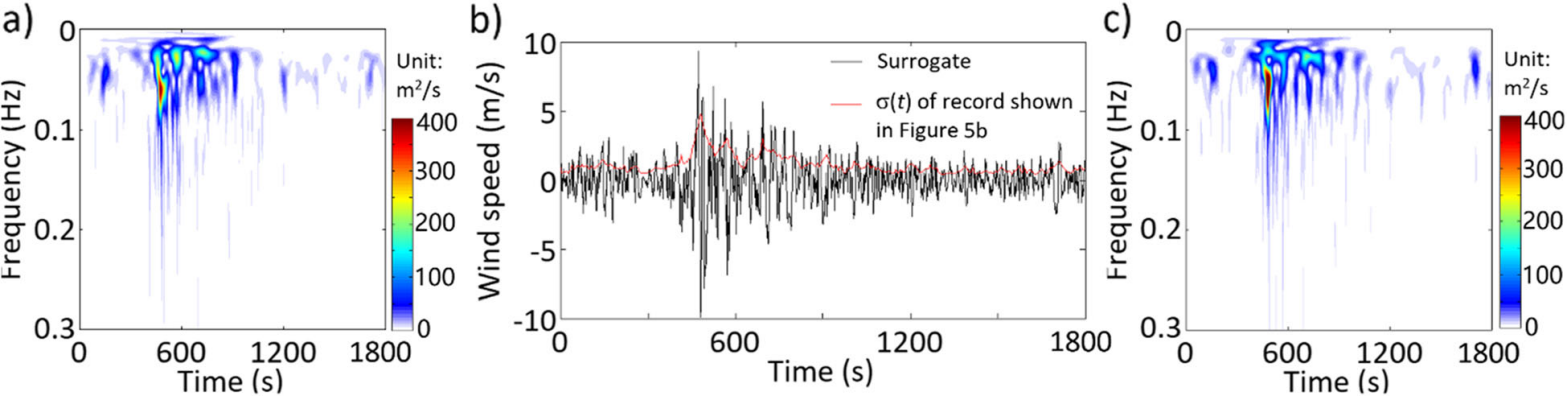
Results by using ST for the wind record shown in Fig. 5b: **a** TFPSD of the given record, **b** a generated surrogate and σ(*t*) of the given record, and **c** TFPSD of the generated surrogate shown in Fig. 6b

**Fig. 7:**
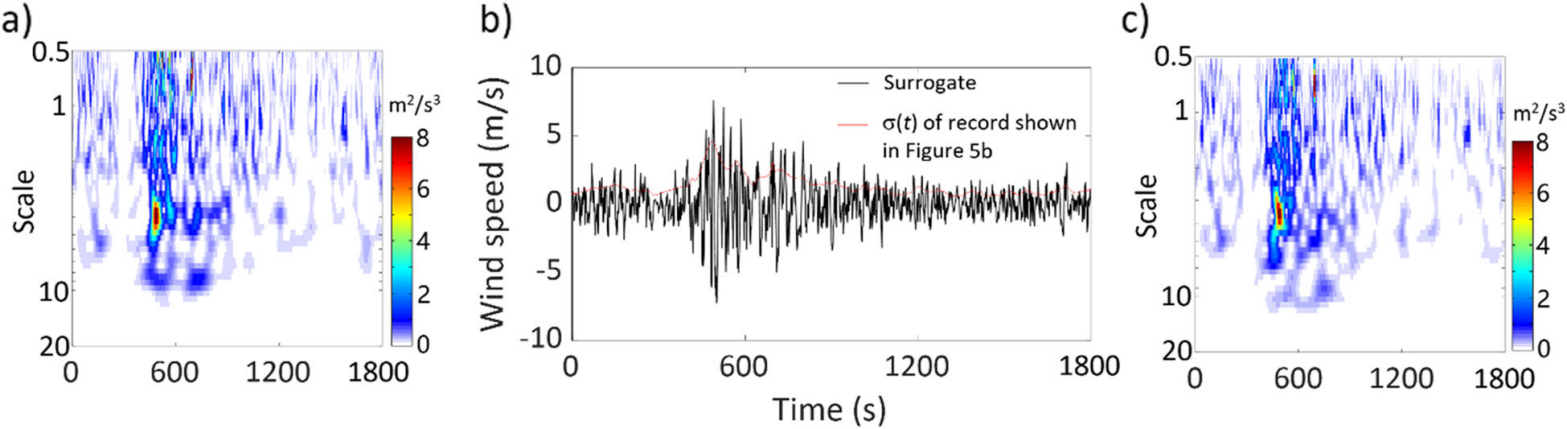
Results by using WT for the wind record shown in Fig. 5b: **a** TSPSD of the given record, **b** a generated surrogate and σ(*t*) of the given record, and **c** TSPSD of the generated surrogate shown in Fig. 7b

By applying ST and WT, and following the same analyses that are carried out for the ground motion record shown in the previous section, the obtained results are presented in Figs. 6 and 7. In general, the observations that can be drawn from this example are similar to those presented in the previous section for the ground motion record.

#### Simulating ground motions

**Fig. 8:**
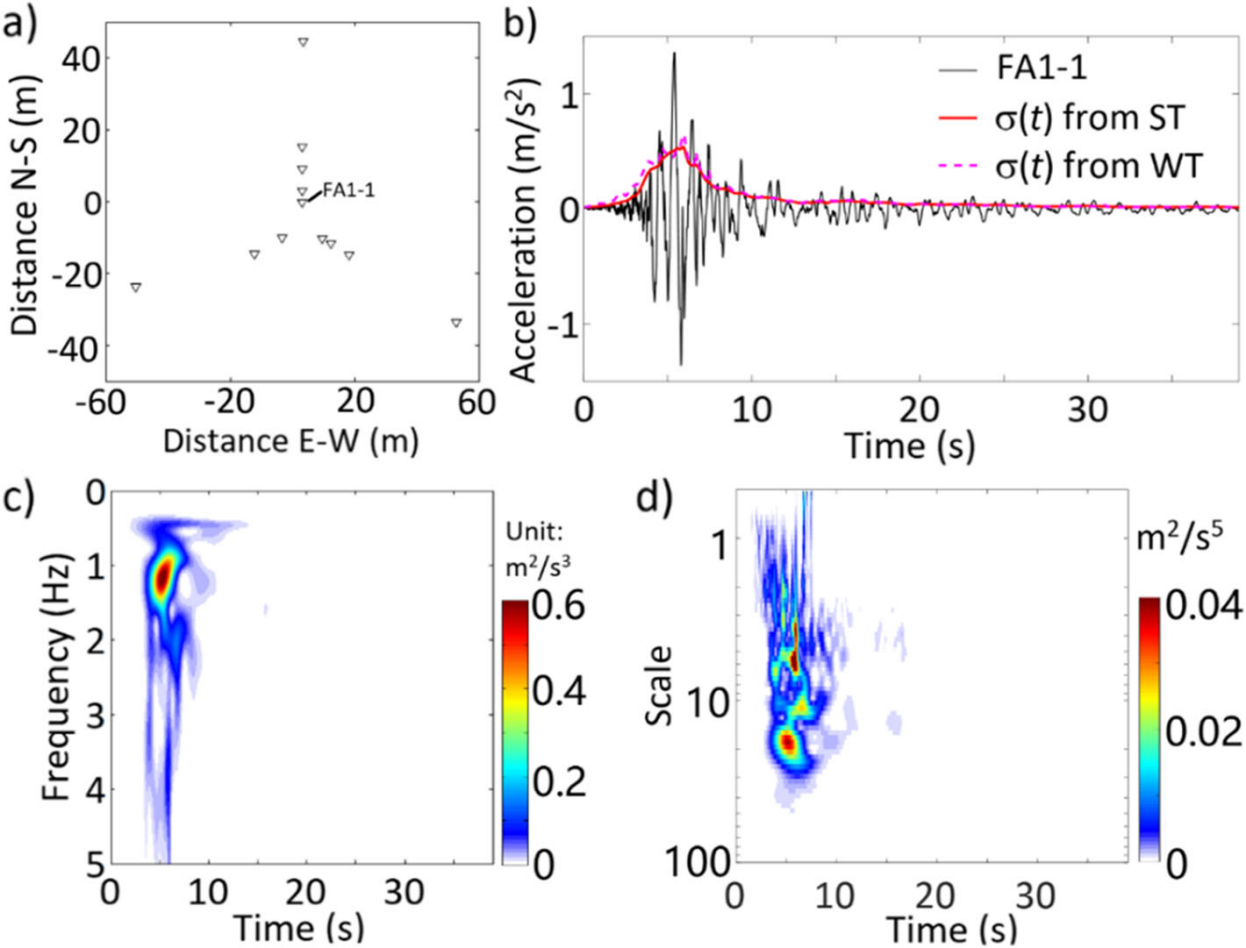
**a** The LSST array station of selected records, **b** record at FA1–1 station, **c** average TFPSD by using ST based on 12 records, and **d** average TSPSD by using WT based 12 records

Consider a set of 12 ground motion records oriented in the E-W direction for a seismic event that occurred on January 16, 1986, with a local magnitude of 6.1, focal depth of 10.2 km, and an epicentral distance of 25.2 km. These records are recorded by the LSST array in Lotung, Taiwan, where the separation between any two recording sites is less than 100 m, as shown in Fig. 8a. Three records from the 15 recording sites seem to be corrupted and are not considered. The record obtained from FA-1 site is illustrated in Fig. 8b. To minimizing the wave passage effect, first, each of the remaining 11 records is time-shifted with respect to the record presented in Fig. 8b such that the sum of the product of a considered record and that shown in Fig. 8b is maximized after the shift.

It is assumed that the average PSD of the considered 12 records could provide a good representation of the ground motions, at least for such type of seismic event. The calculated average TFPSD based on ST and the calculated average TSPSD based on WT are shown in Fig. 8c and d, respectively. The calculated σ(*t*) by using the average TFPSD and the average TSPSD presented in Fig. 8c and d are included in Fig. 8b. The obtained PSD and the standard deviation indicate the nonstationarity of the ground motions. σ(*t*) values obtained by using ST and WT are in very good agreement.

**Fig. 9:**
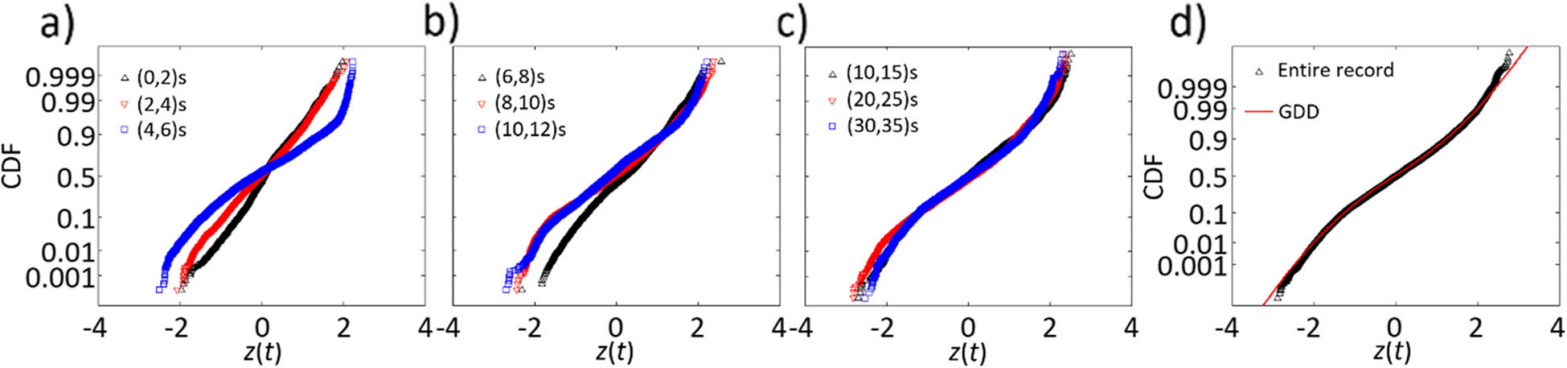
Empirical distributions of the normalized time series of the considered ground motions. **a** Time interval (0, 2), (2, 4), (4, 6); **b** (6, 8), (8,10), (10, 12); **c** (10, 15), (20, 25), (30, 35); **d** entire duration and the fitted GGD with β_0_ = 3.01 and β_1_ = 1.54

An assessment of the empirical probability distribution of the standardized variable *z*(*t*) = *x*(*t*)/*σ*(*t*) is carried out by considering all 12 records. The empirical distribution of *z*(*t*) by considering all 12 records is presented in Fig. 9, indicating that the empirical distribution can be fitted by a normal distribution only for the initial segment of the records. Moreover, the distribution shape is time-varying and non-Gaussian. The non-Gaussian behaviour of the ground motions is supported by the findings given in Radu and Grigoriu [40], indicating that the Gaussian assumption for the seismic ground motions records included in the PEER NGA-West dataset may not always appropriate. However, for this particular set of records, the tail of the distribution is shorter than that of the normal distribution, which differs from the longer tail behaviour suggested by Radu and Grigoriu [40].

For illustration purposes, it is assumed that the marginal probability density function of *x*(*t*) can be represented by the generalized Gaussian distribution (GGD) [29, 54],

31 $$ {f}_{X,t}\left(x(t)\right)=\frac{\beta_0}{2{\beta}_1\Gamma \left(1/{\beta}_0\right)}{e}^{-{\left(\left|x(t)-\mu \right|/{\beta}_1\right)}^{\beta_0}}, $$

where μ denotes the mean, β_0_ and β_1_ are positive model parameters, and Γ(·) denotes the gamma function. If β_0_ equals 2, it represents the normal distribution. For β_0_ > 2 and < 2, the distribution tail is lighter and heavier than that of normal distribution. The variance equals ${\beta}_1^2\Gamma \left(3/{\beta}_0\right)/\Gamma \left(1/{\beta}_0\right)$, and the kurtosis coefficient equals Γ(5/*β*_0_)Γ(1/*β*_0_)/Γ^2^(3/*β*_0_).

**Fig. 10:**
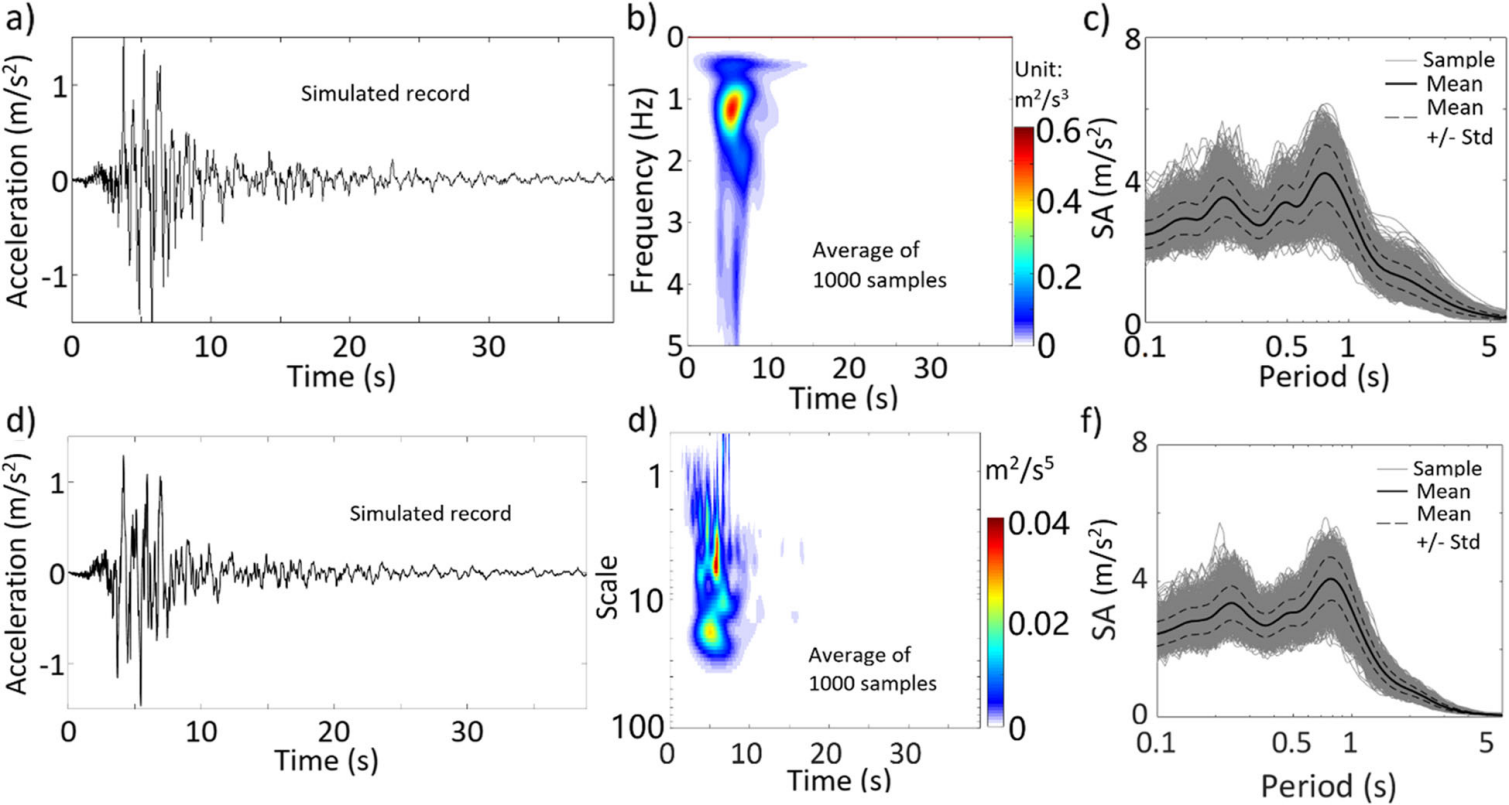
Results based on simulated nonstationary Gaussian records by using ST and WT (the results presented in **a** to **c** are for ST, and the results presented in **d** to **f** are for WT): **a** a sampled record based on ST, **b** average TFPSD of the 1000 sampled record, **c** SA from 1000 sampled records using ST, **d** a sampled record based on WT, **e** average TSPSD of the 1000 sampled record, **f** SA from 1000 sampled records using WT

By considering β_0_ = 2 and ${\beta}_1=\sqrt{2}$ (i.e., standard Gaussian), we use ST and the average target TFPSD function shown in Fig. 8c to sample the records using the IPAC algorithm. Since a comparison of two sampled records is irrelevant for a stochastic process, only a sampled record is illustrated in Fig. 10a. The average TFPSD function obtained from the 1000 sampled records is presented in Fig. 10b, and the calculated spectral acceleration (SA) for a damping ratio of 5% is shown in Fig. 10c for the 1000 sampled records. Similarly, we use WT and the average target TFPSD function shown in Fig. 8d to carry out the simulation. The obtained results are presented in Fig. 10d-f. The PSD functions shown in Fig. 10b and e are almost identical to their corresponding targets presented in Fig. 8c and d. The mean of SA values shown in Fig. 10c and f are in good agreement. The standard deviation of SA obtained by using ST smaller than that obtained by using WT.

We have tested the IPAC algorithm to simulate ground motions for additionally selected target PSD functions. It was observed that in some cases, when the initial or final segment of records has less than 0.5% of total energy, the algorithm may converge very slowly or may not converge. In such a case, it is suggested that such segments with negligible energy are to be truncated. This is in agreement with common practice in earthquake engineering.

**Fig. 11:**
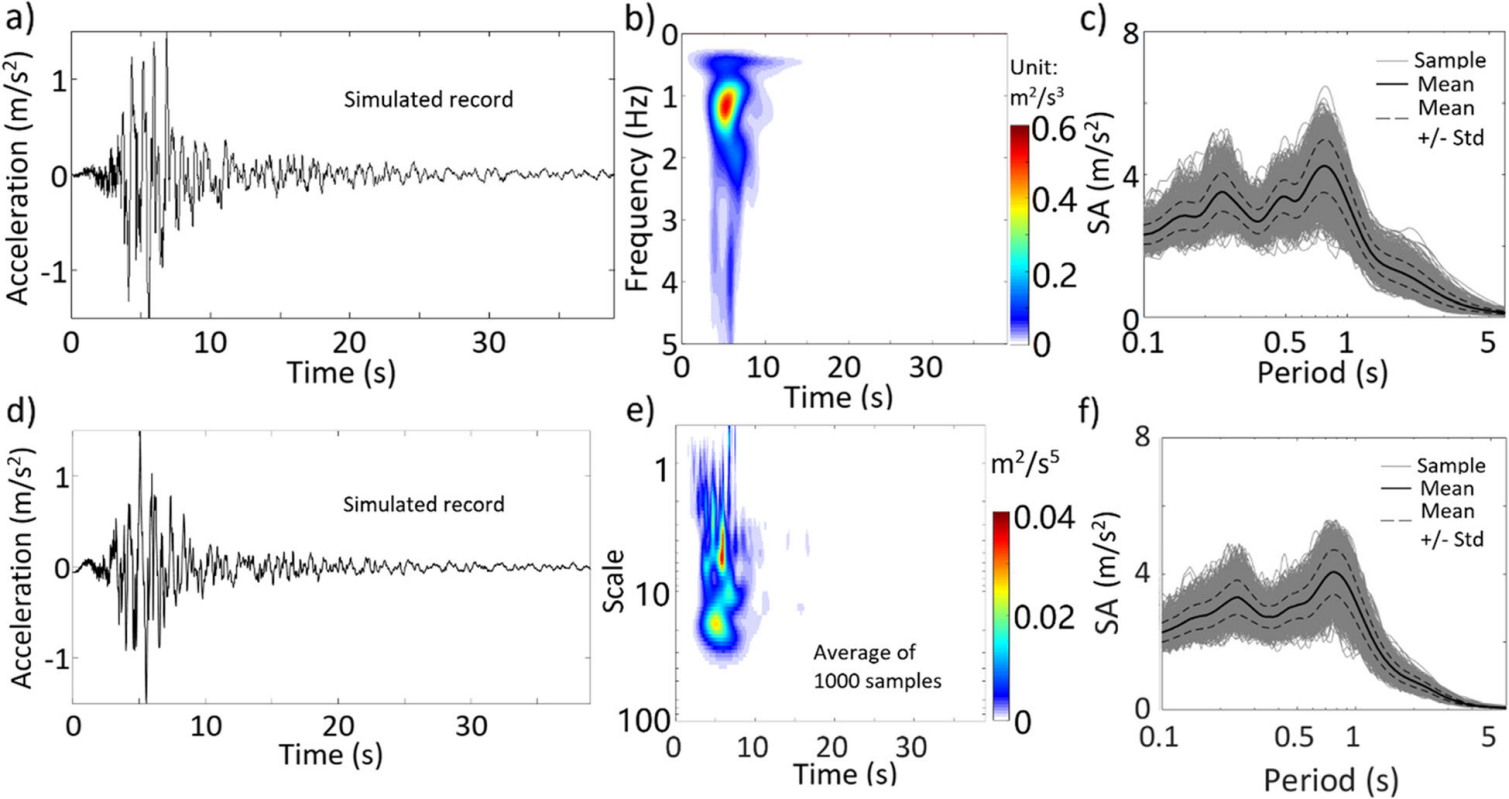
Results based on simulated nonstationary non-Gaussian records by using ST and WT (the results presented in **a** to **c** are for ST, and the results presented in **d** to **f** are for WT): **a** a sampled record based on ST, **b** average TFPSD of the 1000 sampled record, **c** SA from 1000 sampled records using ST, **d** a sampled record based on WT, **e** average TSPSD of the 1000 sampled record, **f** SA from 1000 sampled records using WT

**Fig. 12:**
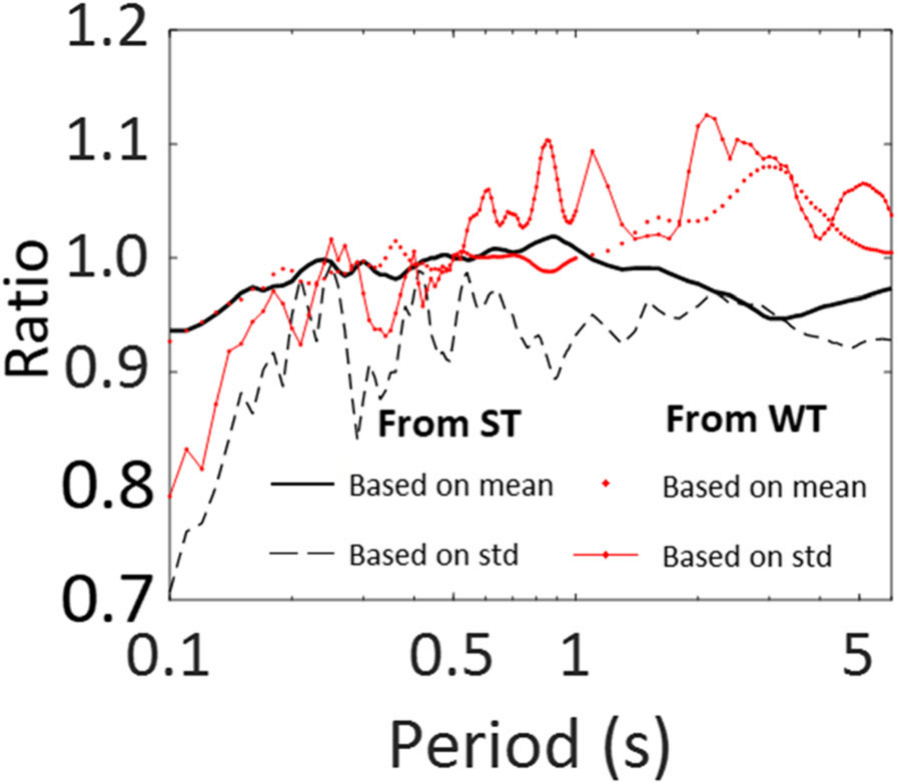
Ratio of statistics of SA of simulated records with non-Gaussian and Gaussian distribution assumptions

To simulate the non-Gaussian process, we consider *f*_*X*, *t*_(*x*(*t*)) shown in Eq. (31) but with β_0_ = 3.01 and β_1_ = 1.54 (i.e., a kurtosis coefficient of 2.4) since their use fit the data adequately, as depicted in the last plot in Fig. 9. We repeat the analysis that is carried out for the results presented in Fig. 10. The obtained results for non-Gaussian ground motions are presented in Fig. 11. A comparison of the results shown in Figs. 10 and 11 indicates that the results follow the same trends. To assess the quantitative differences between the obtained SA based on Gaussian and non-Gaussian assumptions, we calculate the ratio of the mean of SA shown in Fig. 11 (i.e., non-Gaussian case) to its corresponding value shown in Fig. 10 (i.e., Gaussian case). We do the same for the standard deviation of SA. The obtained values are presented in Fig. 12, indicating that the mean and standard deviation of SA for the non-Gaussian case with a lighter tail are smaller than those for the Gaussian case for the vibration period less than about 0.5 s. The decrease in SA by considering non-Gaussian excitation is most noticeable for a shorter vibration period. This is because stiffer structures are more sensitive to peak acceleration values. In general, the ratio based on ST is smoother than that based on WT. Note that we refrained from discussing the ratio between the SA values obtained based on ST and WT since such a comparison could be misleading because the TSPSD and TSPSD used are based only on 12 records and from the same seismic event.

We note that the simulation of a record (Gaussian or non-Gaussian) described in Figs. 10 and 11 is carried out by using a laptop with Intel(R) Core (TM) i7–7700 CPU @ 3.60GHz (6 core and 12 treads). The wall clock time for simulating a record (including I/O) is less than about 0.5 s if ST is used and is less than about 0.15 s if CWT is used. The difference in the computing time by using ST and CWT can be explained by noting that the octave scale is used in CWT.

## Summary and conclusions

We elaborated on the concept of defining a modulated and intensity function adjusted (MODIF) stochastic process in the transform domain. The definitions of the stochastic processes in the transform domain lend themselves to an easily understandable and almost trivial algorithm to simulate stochastic processes. As such a simulated signal may not lead to the prescribed target power spectral density function and marginal cumulative distribution function of the process, we proposed a new iterative algorithm, called iterative power and amplitude correction (IPAC) algorithm, so the sampled signal after the iteration have the prescribed properties. Besides simulating nonstationary and non-Gaussian processes, the proposed iterative algorithm can be used to generate surrogate. The algorithm can be used with Fourier transform, S-transform, and continuous wavelet transforms.

Practical illustrative numerical examples showed the applicability of the proposed algorithm by sampling surrogates for the ground motions and the fluctuating component of winds. The use of the IPAC algorithm to simulate nonstationary Gaussian and non-Gaussian ground motions based on S-transform (ST) and continuous wavelet transform (WT) is presented. The spectral accelerations are calculated using the simulated records. In general, the mean and standard deviation of SA of the simulated records based on ST and based on WT agree well despite the differences between ST and continuous WT and between the frequency-dependent window used in ST and the generalized Morse wavelet used in the continuous WT.

## Data Availability

The ground motion record shown in Fig. 2 is from http://aplicaciones.iingen.unam.mx/AcelerogramasRSM/Inicio.aspx; the 12 ground motion records from LSST array are from http://www.earth.sinica.edu.tw/. Some or all data, models or code generated or used during the study are available from the corresponding author by request. These include all the coefficients of FT, ST, and WT used in the present study and the simulated time histories.

## Abbreviations

CDF: Cumulative distribution function
FT: Fourier transform
FFT: Fast Fourier transform
GMWs: Generalized morse wavelets
GGD: Generalized Gaussian distribution
MODIF: Modulated and intensity function adjusted
IAAFT: Iterative amplitude adjusted Fourier transform
IFT: Inverse Fourier transform
IPAC: Iterative power and amplitude correction
PSD: power spectral density
SA: Spectral acceleration
ST: S-transform
TFPSD: Time-frequency PSD
TSPSD: Time-scale PSD
WT: Wavelet transform
